# Effectiveness, Mediators, and Effect Predictors of Internet Interventions for Chronic Cancer-Related Fatigue: The Design and an Analysis Plan of a 3-Armed Randomized Controlled Trial

**DOI:** 10.2196/resprot.4363

**Published:** 2015-06-23

**Authors:** Marije DJ Wolvers, Fieke Z Bruggeman-Everts, Marije L Van der Lee, Rens Van de Schoot, Miriam MR Vollenbroek-Hutten

**Affiliations:** ^1^ Roessingh Research and Development Telemedicine Group Enschede Netherlands; ^2^ University of Twente Faculty of Electrical Engineering, Mathematics and Computer Science Telemedicine Group Enschede Netherlands; ^3^ Helen Dowling Institute Scientific Research Department Bilthoven Netherlands; ^4^ Utrecht University Department of Methods and Statistics Utrecht Netherlands; ^5^ North-West University Vanderbijlpark South Africa

**Keywords:** fatigue, cancer survivors, chronic disease, Internet interventions, mindfulness-based cognitive therapy, motor activity, behavior therapy, accelerometry, effect predictors, mediation, Bayesian statistics, latent growth analysis

## Abstract

**Background:**

Internet interventions offer advantages that especially cancer survivors who suffer from fatigue could benefit from. Given the growing number of such patients, Internet interventions could supplement and strengthen currently available health care.

**Objective:**

This paper describes the design and analysis plan that will be used to study 2 Internet interventions aimed at reducing severe fatigue in cancer survivors: a mobile ambulant activity feedback therapy supported through a weekly email by a physiotherapist and a weekly Web- and mindfulness-based cognitive therapy supported online by a psychologist. The data resulting from this trial will be used to (1) investigate the effectiveness, (2) investigate potential mediators of these interventions, and (3) explore participant characteristics that can predict the effect of these interventions.

**Methods:**

A 3-armed randomized controlled trial is proposed that compares both Internet interventions with an active control condition that solely consists of receiving psycho-educational emails. The intervention period is 9 weeks for all 3 conditions. Six months after baseline, participants in the control condition can choose to follow 1 of the 2 experimental Internet interventions. Outcomes are measured in terms of fatigue severity, mental health, and self-perceived work ability. All are Web-assessed at baseline, 2 weeks after the intervention period, and at 6 and 12 months after baseline. Fatigue severity, mindfulness, physical activity, expectations and credibility of the intervention, therapeutic working alliance, sleep quality, and sense of control over fatigue are assessed 3 times during the intervention period for identifying mediators of the interventions. Recruitment is performed nationally throughout the Netherlands through patient organizations and their websites, newspapers, and by informing various types of health professionals. All participants register at an open-access website. We aim at including 330 cancer survivors who have finished curative-intent cancer treatment at least 3 months previously, and have been suffering from severe fatigue ever since. All cancer types are included. A detailed analysis plan is described to address the research questions, which allows for individual variation, and fully exploits the longitudinal design.

**Results:**

Recruitment started in April 2013 and will proceed until April 2015.

**Conclusions:**

This paper describes a systematic trial design for studying 2 different interventions for chronic cancer-related fatigue in order to gain insight into the effectiveness and mediators of the interventions. This design will also be used to identify predictors for the interventions’ effect on fatigue. By publishing our hypotheses and analysis plan before completion of data collection, this paper is a first step in reporting on this trial comprehensively.

**Trial Registration:**

The Netherlands National Trial Register (NTR3483). (Archived by WebCite at http://www.webcitation.org/6NWZqon3o).

## Introduction

### Background

Behavioral interventions have shown to effectively relieve psychological and physical complaints in cancer survivors. However, the effect on the individual is less explicit, because patients differ greatly in the ways they experience and respond to such interventions. Therefore, when studying such an intervention, individual differences and temporal aspects need to be appreciated. This paper presents a detailed analysis plan for studying behavioral interventions that satisfies such needs.

The protocol of a 3-armed randomized controlled trial is described to study the effectiveness, mediators, and effect predictors of 2 different Internet interventions that share the same aim: reducing fatigue for cancer survivors. Due to its longitudinal design and multiple assessments during the intervention, the temporal development of relevant factors rather than pre-post differences can be studied. Latent growth analysis can be performed and mixture models can be run, which allow for individual variance in growth trajectories. Furthermore, full longitudinal mediation analyses can be performed on the most important potential mediators of both interventions, and differentiating effect predictors can be identified in order to allocate individuals to the most suitable intervention.

The goal of this paper is to present our trial design, hypotheses, and analysis plan. This paper will therefore be the basis for a number of papers that will present the results of the trial. We will first provide brief background information on the research population, the relevance of Internet interventions for this population, and introduce the 2 Internet interventions that are the subject of this trial. Next, the importance of identifying mediating and predicting factors for the intervention effect is discussed. In the remaining sections, we give a detailed description of the trial’s design, our hypotheses, and the analysis plan for handling the data that the trial will collect. The analysis plan is written in general terms, in order to facilitate the use of this strategy in other contexts, and to keep this paper focused. Consequently, the extended background of—and reasoning for—the specific hypotheses will be presented in future papers that will focus on the results of the proposed analyses.

### Chronic Fatigue and Cancer

Cancer-related fatigue is defined as “a persistent, subjective sense of physical, emotional and/or cognitive tiredness or exhaustion related to cancer or cancer treatment that is not proportional to recent activity, and interferes with usual functioning” [1]. It is 1 of the most prevalent and distressing long-term consequences of cancer [2], interferes with the activities of daily living, work ability [3], and maintenance of social relations, and consequently impacts patients’ well-being [4]. As the number of cancer survivors in the Netherlands is expected to increase rapidly, with a growth of over 50% in the 10-year prevalence between 2009 and 2020 [5], there is a strong need for effective and accessible treatments.

The etiology of cancer-related fatigue probably involves the deregulation of several interrelated physiological, biochemical, and psychological systems [6]. There is no definite somatic explanation for the persistence of fatigue after cancer [7-9], and estimates of the proportion of cancer survivors who suffer from persistent fatigue vary widely [8,10]. However, research has shown that if fatigue continues 3 months after treatment, it is unlikely to decrease of its own accord [8]. The term chronic cancer-related fatigue (CCRF) is used in this paper for severe fatigue that continues for 3 months or longer after cancer treatment completion.

### Management of Chronic Cancer-Related Fatigue

Currently, both pharmacological treatments and nonpharmacological treatments are applied to the effective management of CCRF; see the overview articles published by Ahlberg et al [9] and Koornstra et al [11]. Guidelines state that if no primary association can be found for the persistence of fatigue with a somatic condition, behavioral interventions should also be considered [1]. The previously reported effects of nonpharmacological interventions on fatigue vary widely, as can be seen in the overview of recent meta-analyses in Table 1. Effect sizes tend to be higher when the intervention targets fatigue, and when increased fatigue was an inclusion criterion for the study. Not all studies that were included in the meta-analyses primarily targeted fatigue, therefore effect sizes might not be representative for nonpharmacological interventions that target fatigue.

**Table 1:** Ten recent meta-analyses considering nonpharmacological interventions for cancer patients that included off-treatment fatigue.

Meta-analyses (off treatment)	Intervention type	ES^a^ (95% CI)	Fatigue reduced (*P*-value)
Jacobsen 2007 (k^b^=4%)	Psychological	d^c^ = 0.10 (0.02-0.18)	Yes (<.05) [12]
Jacobsen 2007 (k=29%)	Activity-based	d=0.05 (-0.08-0.19)	ns [12]
Kangas 2008 (100%)	Psychological	WMES^d^(r^e^) = 0.51 (0.10-0.92)	Yes (.015) [13]
Kangas 2008 (100%)	Exercise	WMES(r) = 0.13 (-0.77-1.02)	ns (.784) [13]
Speck 2010 (100%)	Exercise	WMES(r) = 0.54 (0.19-0.90)	Yes (.003) [14]
Brown 2011 (k=54%)	Exercise	WMES(r) = 0.31 (0.22-0.40)	Yes [15]
Duijts 2011 (n=31%)	Behavioral techniques	SMD^f^(f^g^) = 0.16 (0.08-0.23)	Yes (<.001) [16]
Duijts 2011 (n^h^=42%)	Exercise	SMD(r) = 0.315 (0.10-0.53)	Yes (.004) [16]
Cramp 2012 (100%)	Exercise	SMD = 0.37 (0.18-0.55)	Yes [17]
Tomlinson 2014 (100%)	Exercise	SMD(r) = 0.61 (0.33-0.88)	Yes [18]

^a^ES = Effect size; values are positive when the intervention was able to reduce fatigue more compared to the control condition.

^b^k=percentage of studies

^c^d=Cohen’s d

^d^WMES = weighted means effect size

^e^r=random effects

^f^SMD = standardized mean difference

^g^f=fixed effects

^h^n=percentage of participants

Behavioral interventions are often based on energy balance models and/or stress coping models [12,19]. In energy balance models, CCRF is seen as a consequence of deconditioning and prolonged inactivity during cancer and its treatment. Secondary fatigue arises as a result of detraining and can lead to a downward spiral. In stress coping models, CCRF is conceptualized as a result of ineffective coping strategies and prolonged stress response [20]. Cognitive behavioral treatments that are based on these theories include physical activity interventions, exercise interventions [14,17,21,15,22], and mindfulness-based cognitive interventions [23-26] and have been shown to help reduce CCRF in previous studies [12,13]. However, all these interventions require the patient to travel to a health care facility, which can be a burden to the patient. Therefore, introducing effective interventions in a home-based setting could improve the health care options for this group.

### Potential Benefits of Internet Interventions

Internet interventions offer advantages that cancer survivors who suffer from fatigue could especially benefit from. They have been found to be as effective as face-to-face therapies for a wide range of disorders, such as posttraumatic stress disorder, burnout or chronic stress, and depression [27-32]. Internet interventions have the ability to reach a wider range of patients compared to face-to-face interventions, especially severely fatigued patients, those with limited mobility, or patients in rural or even remote areas. Also, patients may benefit from the home-based setting of Internet interventions as these patients can practice more often, are less bound to the availability of care professionals, and can incorporate the intended behavioral change directly into their daily routine. Moreover, visiting a health care facility may no longer be desirable for some cancer survivors due to negative associations with the disease process or because they no longer want to be identified as a cancer patient and prefer the anonymity of their own environment.

### Internet Interventions for Fatigue

#### Overview

In the Netherlands, to our best knowledge there are currently 3 Internet interventions that aim to reduce chronic fatigue: (1) an experimental mobile intervention aimed at changing physical activity behavior for participants with chronic fatigue syndrome [33]; (2) the Web-based mindfulness-based cognitive therapy “Minder Moe Bij Kanker” [34]; and (3) a Web-based cognitive behavior therapy for severely fatigued breast cancer survivors, which is the subject of the current CHANGE study (trial registration NTR4309) [35].

This paper describes the design and analysis plan that studies the first 2 of these Internet interventions in a randomized controlled trial. Each of these 2 interventions is described below.

#### Mobile Activity Management Intervention: Ambulant Activity Feedback Therapy

The ambulant activity feedback therapy (AAF) is a mobile intervention that utilizes an ambulant activity coaching system, supported weekly by a physiotherapist through email [33]. The activity coaching system was developed by Roessingh Research and Development (Enschede, The Netherlands) and consists of a mobile phone and an accelerometer (Multimedia Appendix 1) that communicate through Bluetooth [33].

**Figure 1:**
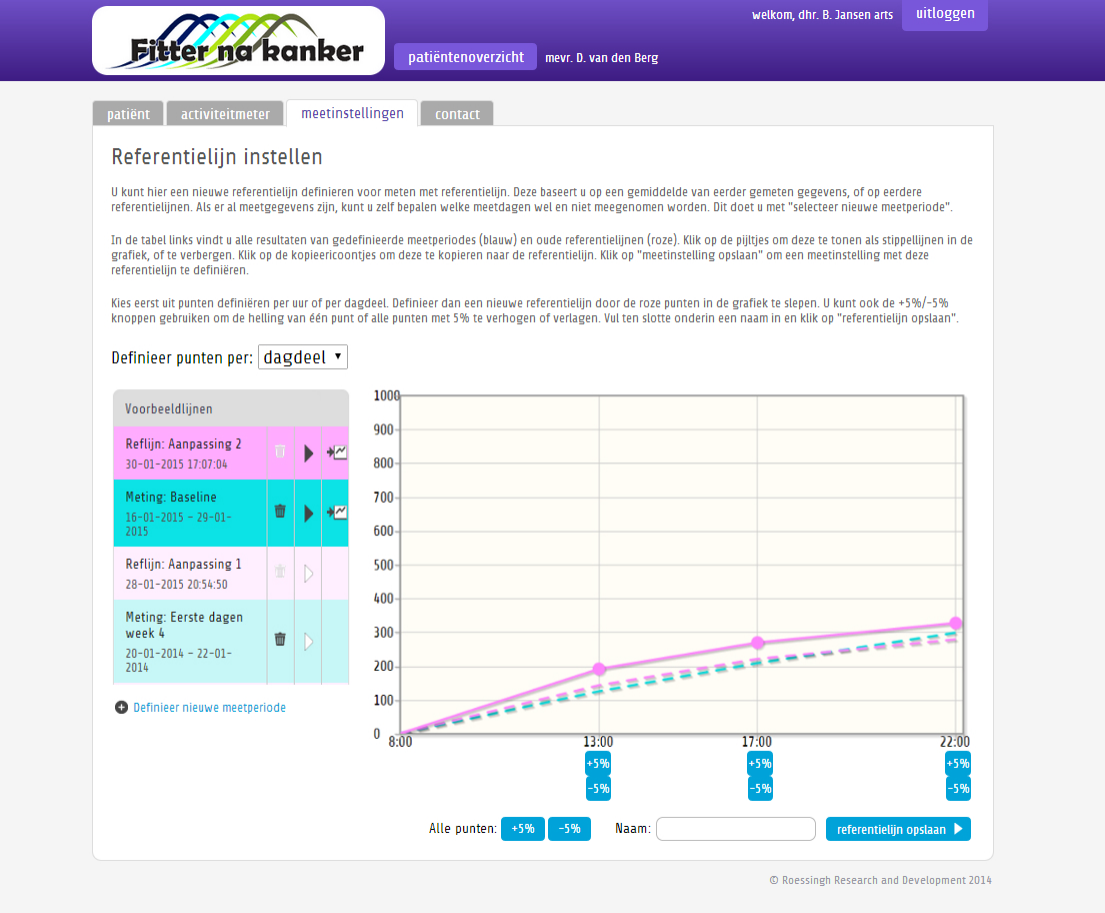
Screenshot of the Web portal for the ambulant activity feedback therapy (Dutch).

In this intervention, the patient works to meet personal activity goals and subgoals that will be defined together with the therapist. The coaching system supports this process by showing real-time feedback about the accumulated activity of the patient relative to a personalized line of reference and tailored hourly feedback messages. Both the line of reference and the set of feedback messages of the activity coaching system can be adjusted by the therapist through a Web portal (see Figure 1 and Multimedia Appendix 2). Patients also have access to a Web portal where they can monitor their past personal activity records. Consequently, patients are expected to gain insight in their activity pattern and on how to increase or balance their daily activity in a way that improves their energy levels. More information about AAF is given in Wolvers and Vollenbroek-Hutten (in press) [36].

#### Web-Based Mindfulness-Based Cognitive Therapy

**Figure 2:**
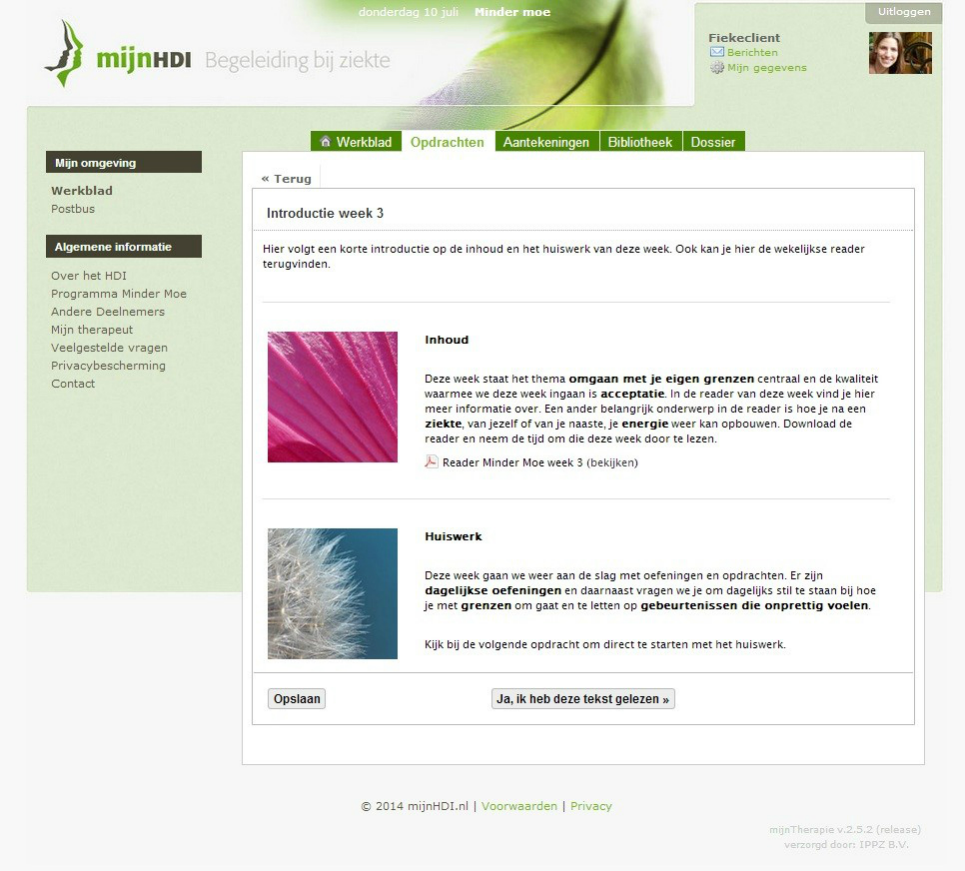
Screenshot of the Web portal for eMBCT (Dutch).

Mindfulness-based cognitive therapy (MBCT) [37] adds elements of cognitive therapy to the mindfulness-based stress reduction program that was originally developed by John Kabat-Zinn [38]. The Helen Dowling Institute (Bilthoven, the Netherlands) developed a 9-week Web-based, therapist-guided, individual MBCT (eMBCT) specifically designed to reduce cancer-related fatigue [23]. On a personal Web page (see Figure 2 and Multimedia Appendix 3), each patient can download audio files of mindfulness exercises and read information about a specific mindfulness theme each week. Patients write down their experiences of following the mindfulness exercises in a log. On an agreed-upon day of the week, the therapist replies to this log, thereby guiding the patient through the program. It is hypothesized that by learning to raise awareness of their present experience nonjudgmentally and openly, the patient can become aware of potentially ineffective coping strategies that prolong stress and fatigue [39,40]. Patients learn to use a detached perspective as a skill to prevent the escalation of automatic negative thinking patterns. MBCT also teaches patients how to accept fatigue, physical limitations, or pain. The protocol of the eMBCT is discussed more extensively in the article by Bruggeman-Everts et al [34].

### Effectiveness

#### Overview

Our primary question is whether both interventions are effective in reducing fatigue. Therefore, the interventions will be compared to an active control group in a randomized controlled trial. The advantage of this design, as compared to a waiting-list control group, is that we can control for nonspecific influences of the trial, such as receiving attention. Also, we expect that in an active control group, fewer participants will drop out than in a waiting-list control group.

Usually, results of interventions are presented in terms of an average improvement of the relevant outcome measure. However, practice shows that individuals benefit differently from interventions [41]. Therefore, the proposed trial will aim to identify individual fatigue trajectories, since that seems to be more informative and helpful in improving health care provisions for CCRF-patients than just presenting averages.

#### Mediators

To optimize interventions in terms of efficiency and effectiveness, treatment-specific and nonspecific working mechanisms should be identified that account for each intervention’s effect on fatigue. Knowledge about these mechanisms is an important prerequisite for improving the efficiency of interventions by shifting focus or shortening the intervention. Also, effectiveness can be increased by improving and tailoring the relevant items, subjects, or exercises, as well as improving the way these are embedded in the intervention. Therefore, the second objective of this study will be to identify the working mechanisms underpinning the interventions.

By using a 3-armed randomized design, it is possible to study both treatment-specific (differentiating) and nonspecific working mechanisms. Also, by assessing important factors multiple times during the intervention, important time-specific information can be acquired.

#### Effect Predictors

Although we expect that, in general, both interventions are effective, personal factors, medical factors, and demographics may determine the effect that each intervention has on fatigue [41]. We do not expect all individuals to benefit similarly from the interventions. Therefore, studying potential predictors of each intervention’s effect will give us important input to inform both patients and caregivers and allow them to set reasonable expectations.

CCRF has a multifactorial character (eg, physical, cognitive, motivation); therefore, studying the effect predictors of 2 theoretically differing interventions simultaneously might also reveal differentiating predictors for both therapies. By applying such knowledge carefully, the overall effectiveness of interventions that aim to reduce CCRF can be increased.

## Methods

### Design

**Figure 3:**
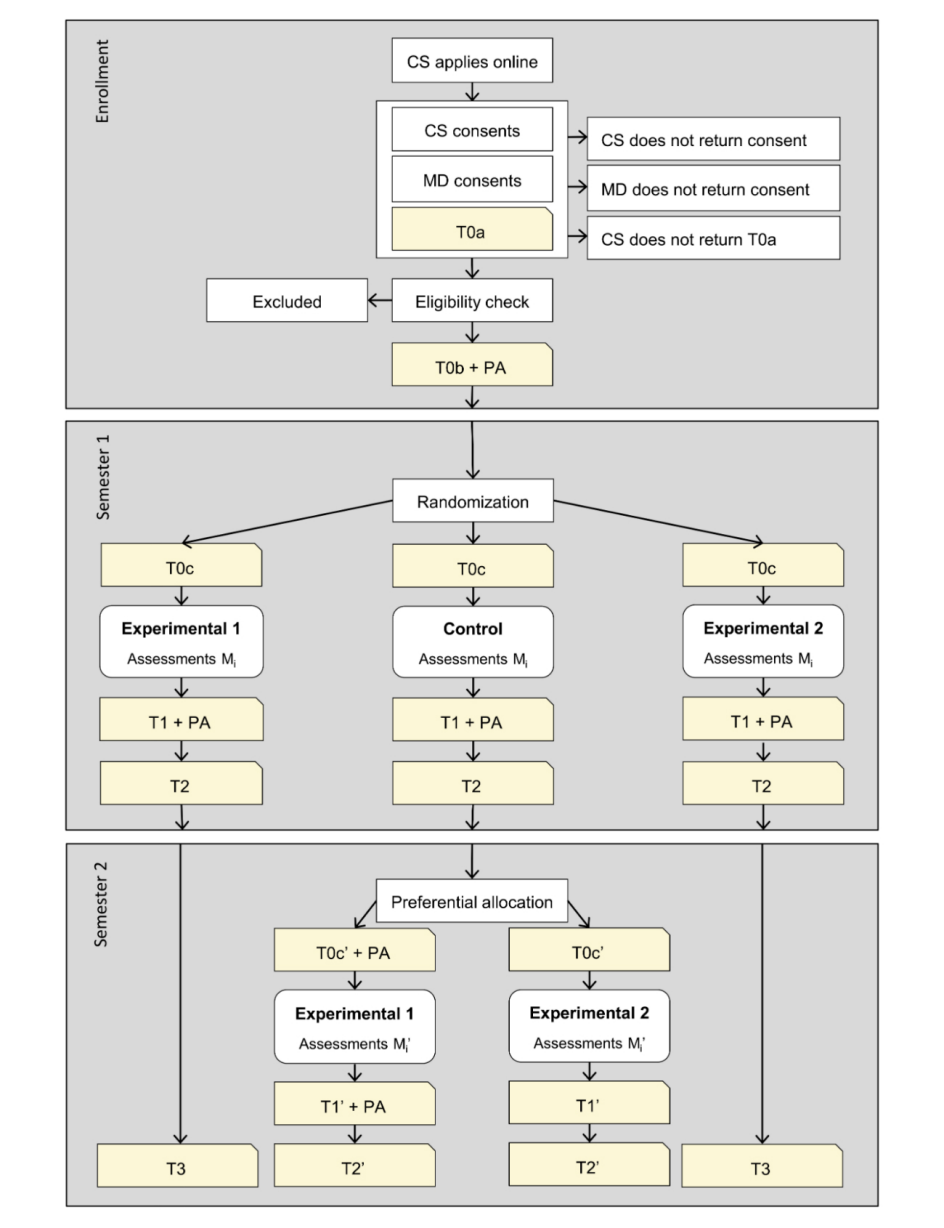
Flow chart of trial design. CS = cancer survivor; MD = medical doctor; PA = physical activity; T0(a-c)-T3 are the main assessments; Mi and Mi' represent assessments in week (i =) 1, 2, 3, 4, 6, and 9 of the intervention. For addressing the primary research questions of effectiveness, only data from the first semester will be used.

A randomized controlled trial is performed including 3 parallel conditions: 2 experimental conditions (AAF and eMBCT) and a minimal intervention control condition. The intervention period is 9 weeks for all 3 conditions. Both experimental conditions are made as similar as possible in terms of time-investment and contact intensity with the therapist. Outcomes are self-reported and are Web-assessed at baseline (T0), 2 weeks post-intervention (T1), and at 6 months (T2) and 12 months (T3) after baseline. Figure 3 shows a schematic summary of the trial design.

The baseline assessment consists of 3 time-points: (1) T0a, the assessment to check eligibility; (2) T0b, the main baseline assessment taken after the eligibility check and informed consent, but naive of condition; and (3) T0c, directly after randomization for assessing the participant’s credibility and expectancy about the condition. All participants are invited to fill out short questionnaires in weeks 1, 2, 3, 4, 6, and 9 (Mi) of the intervention period in order to study mediation of the interventions.

After T2, patients in the control condition are offered 1 of the 2 experimental interventions, again in a research setting. Please note that the first 4 participants of this trial were randomized to 1 of the experimental conditions for the second semester, but to minimize dropout, all other patients will be allocated based on their own preference. During this second intervention period, these participants will again be assessed in weeks 1, 2, 3, 4, 6, and 9 (Mi’), the second week after the intervention (T1’), and 6 months after the second allocation (T2’). Participants in the control condition that are preferentially allocated to eMBCT after T2 do not wear the accelerometer during the second semester.

The protocol allows delay within the intervention period of a maximum of 2 weeks in case of, for example, illness or holiday. For all participants, the duration of their participation is approximately 12 months. Additional qualitative feedback will be obtained through explorative interviews with a subset of participants in the experimental condition shortly after T1 or T1’.

This trial was approved by the Twente Medical Ethical Committee (Enschede, the Netherlands) under number P12-26 and has been registered at The Netherlands National Trial Register under number NTR3483 [42].

### Study Sample

#### Recruitment

Participants are recruited in the Netherlands by advertisements in the newsletters of patient associations (both digital and print), on relevant websites, in regional newspapers, and through social media. Furthermore, participants also are recruited through oral presentations given to cancer patients and in other cancer-related seminars and symposia for patients, caregivers, or both.

Social media and online advertising can be strong tools for reaching a large group of people [43] or even specific patients [44,45]. However, the sample might be younger, more highly educated, and might comprise more females compared to the Dutch CCRF population [46]. However, it will lead to a sample that represents the targeted population for such Internet interventions, and we are likely to include participants who would not have opted for therapy that includes traveling to a health care facility.

#### Eligibility

The following criteria are used to check eligibility for participation in the trial:

- Completion of a curative-intent treatment for cancer at least 3 months ago (checked by participant’s medical doctor). For this study, surgery, chemotherapy, radiotherapy, immunotherapy, and/or stem cell transplantation are considered treatment. However, hormonal therapy, the use of anti-inflammatories, and monitoring visits are not considered treatment for this study.
- Patient has been suffering from severe fatigue for at least 3 months.
- Patient scores 35 or higher on the fatigue severity subscale of the Checklist for Individual Strength (CIS).
- Aged 19 years old or older.
- At least 18 years old at disease onset.
- Capable of reading and writing in the Dutch language and of using the Internet (implicit eligibility criterion accounted for during registration, but not checked explicitly).

If patients meet 1 or more of the following criteria, they are excluded from participation:

- Indication of current disease or tumor activity (checked by the participant’s medical doctor).
- Current or former severe psychiatric morbidity, for example, major depression, psychosis, or schizophrenia (checked by the participant’s medical doctor).
- Being dependent on a wheelchair for daily activity (self-report).
- Recurrence of cancer during the course of the study (self-report).
- Current substance abuse, except for smoking.
- Previously attended the eMBCT of the Helen Dowling Institute.

In addition to the mentioned exclusion criteria, please note that:

- Mild depression is not an exclusion criterion. A score of 20 points or higher on the Hospital Anxiety and Depression Scale (HADS) during baseline is considered indicative of depression [47]. Therefore, if the patient scores 20 points or higher, he or she will be contacted by a psychologist from the Helen Dowling Institute to determine whether the participant has suicidal ideation or suffers from other severe psychiatric morbidity. A participant will only be excluded if, according to the involved psychologist, that is the case.
- Comorbid somatic diseases—such as cardiovascular diseases, cerebrovascular diseases, diabetes, hypertension, and arthritis that are not treatable but are a possible cause of fatigue—are not exclusion criteria but will be registered during the study. Although this choice will probably lead to an underestimated effect size compared to studies that do exclude patients with comorbidities, we expect that such a sample will lead to a better representation of the CCRF population.
- Participants are requested not to take part in any other therapy directed at overcoming fatigue during the study.
- Data of participants who report pregnancy or recurrence of cancer during the course of the study will be excluded from analysis since the fatigue they experience cannot be considered to be of a chronic character according to our definitions. However, if requested, these patients will be allowed to finish the intervention.

### Procedures

Participants apply for inclusion in the study at the project website [48,49].

#### Informed Consent

After online registration, participants receive the patient information and informed consent form by direct mail. They are requested to sign and return the informed consent in a prepaid envelope. Also, they receive a registration confirmation by email with login details for the participant’s Web portal on the project website. Participants are requested to complete assessment T0a as a check on eligibility. Also, the participant’s medical doctor is consulted to check 3 of the eligibility criteria: finished curative-intent treatment for cancer more than 3 months ago, no current signs of cancer activity, absence of current or former major psychiatric disease.

#### Randomization

If the eligibility-criteria are met, the researcher confirms the participant’s enrollment. Subsequently, the activity sensor is given to the participant and its setup is explained in a face-to-face meeting in the participant’s home or another mutually convenient location. The second baseline assessment starts (T0b), followed by randomization of the participant to 1 of the 3 conditions by a script embedded in the researchers’ Web portal and uses the random function of php (rand(1,3)) [50]. The researchers can neither influence nor predict the outcome of the randomization process. Subsequently, the researcher emails the participant about the outcome of randomization, requests the participant to complete the third baseline assessment (T0c), and assigns the participant to a therapist in case the participant has been randomized to an experimental condition. Participants who do not fill out T0c are considered as not being included. The allocation of a therapist is based on current availability of the therapists who are involved in the trial.

#### Research Conditions

Both experimental conditions are described in the Introduction and will be described more extensively in an article on eMBCT by Bruggeman-Everts et al [34], and a paper on the development of the AAF intervention by Wolvers and Vollenbroek-Hutten (in press) [36].

#### Active Control Condition

Patients who are assigned to the control condition receive weekly emails containing standard psycho-educational texts about CCRF in order to minimize the dropout rate, following the design of Postel et al [51]. An example of the information that is offered in this minimal intervention control condition is given in Multimedia Appendix 4 and overlaps completely with the information that is given during both experimental interventions. This condition controls for receiving information on CCRF and for being involved in eHealth research.

#### Nonadherence and Withdrawal

Participants who do not adhere to, or withdraw from, the study or the intervention are contacted by phone and asked for the reason for nonadherence or withdrawal. Participants who want to stop with the intervention are asked to complete a post-intervention assessment at T1 and follow-up assessments at T2 and T3. Participants who withdraw from the study are asked to answer the questions of the fatigue severity subscale of the CIS online or during a telephone conversation.

### Assessments

All self-reported questionnaires are Web-assessed via a Web portal on the project website [48,49], developed by Roessingh Research and Development. Participants receive an email when an assessment becomes available and can log in to the Web portal to complete the questionnaires. During the intervention period, each assessment is available for 1 week, but can stay open longer if therapy is postponed due to, for example, illness or holiday. If a participant has not completed it within 6 days, he or she is reminded by email at least once to complete the questionnaire. Within each assessment, the questionnaires are grouped on the basis of importance and subject. Item sequences of the questionnaires for the mediating factors and outcome measures differ between the assessments. Personal data is stored separately from the research data. An overview of all the assessments is shown in Tables 2 and 3.

Physical activity data is collected using the same device as that used for the ambulant activity feedback therapy: a 3D-accelerometer (ProMove 3D) combined with a mobile phone that collects the accelerometer data and sends it to a secured Web server at Roessingh Research and Development [52]. However, the mobile phone does not give feedback on activity, but does state whether the system is working properly and sends an error message if the connection to the sensor fails. Participants are reminded by email to wear the accelerometer on the day before the start of the week in which they will be using it.

### Outcome Measures

#### Fatigue

Fatigue severity will be assessed with the CIS, which consists of 20 items that score on a 7-point Likert scale [53,54]. The CIS has 4 subscales (fatigue severity, motivation, concentration, and physical fatigue) of which the fatigue severity subscale will be used as the primary outcome (8 items, range: 8-56 points). The CIS has shown good discriminative validity in a working population [55], is sensitive to changes in the chronic fatigue syndrome population [56], and has previously been used with cancer survivors [7,57]. The CIS strongly resembles the Multidimensional Fatigue Inventory, which is often used in international studies [54]. Fatigue severity will be assessed at T0a, T0b, M3, M6, M9, T1, T2, and T3. However, the other 3 subscales will only be assessed at T0b, T1, T2, and T3.

#### Mental Health

Mental health will be assessed from the results of 2 questionnaires: the Positive and Negative Affect Scale (PANAS [52]) and the HADS [58], both of which are included in an item bank for cancer survivors [59]. The PANAS consists of 20 items that score on a 5-point Likert scale and has 2 subscales: positive and negative affect. The HADS consists of 14 items on a 4-point scale, has been validated for a Dutch-speaking population [60], and has previously been used to assess psychological distress in cancer patients [61]. Mental health will be assessed at T0a, T1, T2, and T3.

#### Perceived Ability to Work

The work ability score, which is assessed with the first question of the work ability index [62,63], will also be used as an outcome parameter. It asks: “Imagine that your working ability in the best period of your life is rated 10 points. How would you rate your working ability at the present moment?” It is assessed at T0b, T1, T2, and T3.

Working hours and the level of absenteeism are assessed with questions from the Trimbos and iMTA questionnaire on costs associated with psychiatric illness (TiC-P) [64]). These will be assessed at T0b, T2, and T3.

**Table 2:** Assessments of outcome measures and potentially mediating factors.

Outcome Measures and Potentially Mediating Factors		Parameters	T0^a^	Mi/Mi’^b^	T1/T1’	T2/T2’	T3
Primary outcome	Fatigue severity	CIS fatigue severity subscale: 8 items on a 7-point Likert scale.	a, b	3,6,9	x	x	x
Secondary outcomes	Other dimensions of fatigue	CIS physical and cognitive fatigue and motivation subscales: 4 items for each subscale, all on a 7-point Likert scale.	b		x	x	x
	Affect	PANAS: 20 items on a 5-point Likert scale.	a		x	x	x
	Psychological distress	HADS: 14 items on a 4-point scale.	a		x	x	x
	Self-perceived ability to work	Work ability score: 1 item on a 0-10 numeric rating scale (NRS).	b		x	x	x
	Return to work and working hours	Adapted questions of the TiC-P.	b			x	x
Primary mediating factors	Mindfulness	Freiburg Mindfulness Inventory short form [65,66]: 14 items on a 4-point Likert scale.	b	3,6,9	x	x	x
	Physical activity (PA)	Accelerometer: ProMove 3D [52]. Both summative PA and daily PA decline will be considered.	b	3,6,9^c^	x		
	Sleep quality	Subjective Sleep Quality Scale [67]:15 items (yes/no), and 1 self-conceptualized item (yes/no) that translates into: “Did you use sleep medication?”	b	3,6,9	x	x	x
	Sense of control over fatigue	Self-Efficacy Scale [56]: 7 items on a 4-point Likert scale.	b	3,6,9	x	x	x
	Credibility and expectancy	Credibility and Expectancy Questionnaire [68]: 6 items of which 4 are on a 9-point Likert scale, and 2 items on a 0-100 NRS.	c, c’	1,2,4			
	Working alliance	Working Alliance Inventory short form [69,70]: 12 items on 5-point scale; subscales: goal, task, bond.		1,2,4			
Secondary mediating factors	Perceived physical activity	Four self-conceptualized questions on perceived activity volume, comparative volume, and satisfaction with volume.	b		x	x	x
	Self-efficacy on activities	Selected items from the self-efficacy scales of Bandura [71] and Rodgers [72,73]: 13 items on a 0-100 NRS; subscales: planning and coping.	b		x		
	Catastrophizing	Fatigue Catastrophizing Scale [74,75] 9 selected items on a 5-point Likert scale.	b		x		
	Fear of cancer recurrence	Two selected items on a 7-point Likert scale [76].	b		x		
	Causal attributions	One self-conceptualized open answer question that translates to: “What do you consider as the cause of your fatigue?”	b		x		

^a^Baseline assessment T0 consists of 3 time-points: T0a: before eligibility check, T0b: after inclusion, and T0c: after randomization. T0c’ is assessed after preferential allocation.

^b^Mi and Mi’ = assessments at week (i=) 1, 2, 3, 4, 6, 9 of the intervention

^c^All physical activity measurements are blind except for the experimental activity feedback condition at M3, M6, and M9. In the second semester, M3’, M6’, and M9’ do not include a physical activity measurement in the mindfulness condition.

**Table 3:** Other assessments.

Demographics, medical history, and control factors	T0^a^	T1/T1’	T2/T2’	T3
Age, gender, education, family status, nationality, time since diagnosis, time since previous treatment, fatigue duration, psychological counseling in the past, comorbidity.	a			
Cancer type, cancer treatment, perceived life threat of cancer (7-point Likert scale), known heredity of cancer (yes/no/don’t know), former experience with attention focusing exercise (yes/no), religious beliefs, perceived social support (Multidimensional Scale of Perceived Social Support [77]: 12 items on a 7-point Likert scale).	b			
Medication use, substance use (caffeine, nicotine, alcohol, drugs), quality of life (1 item on a 0-10 NRS).	a	x	x	x
Pain intensity and limitations by pain (2 items on a 7-point Likert scale), body mass index.	b	x	x	x
Life events since previous assessment, professional help received for fatigue outside the scope of the study protocol.		x	x	x
Perceived effectiveness of the intervention (5 items of which 1 item 0-10 NRS and 2 yes/no questions), perceived social support in following the intervention (1-10 NRS).		x		
Social desirability: 6 selected items from the Balanced Inventory of Desirable Responding [78] on 5-point Likert scale.			x	

^a^Baseline assessment T0 consists of 3 time-points: T0a: before eligibility check; T0b: after inclusion; and T0c: after randomization.

#### Mediating Factors

Several categories of mediators will be considered: intervention-specific mediators for either eMBCT (eg, mindfulness, catastrophizing, and fear of cancer recurrence) or AAF (eg, physical activity, perceived physical activity, and self-efficacy on physical activity), and generic mediators (eg, sleep quality, sense of control over fatigue, credibility, expectancy, working alliance, and causal attributions). Furthermore, a distinction is made between primary and secondary mediating factors: primary factors are assessed at multiple occasions during the intervention in order to study the timely development of those factors; secondary factors are not assessed during the intervention. A complete overview of all assessments on mediating factors is given in Table 2.

#### Demographics, Medical History, and Control Factors

Several other factors are assessed, including demographics, medical history, and control factors. All are listed in Table 3.

### Analysis Plan

#### Overview

SPSS software will be used for data management and Mplus [79], which is a latent variable modeling program, for the subsequent analyses. The exact versions of the software used will be reported in the future papers.

#### Pre-Analysis

##### Power Analyses

The sample size for analyses for data relating to the primary objective has been calculated for a repeated measures analysis of variance: based on an alpha of .05, a minimal detectable effect size of f2=.15, and a power of .80, a total number of 55 participants [80] is required in each group to answer the primary research question of this study in a statistically valid manner.

We expect to be able to include 330 eligible participants within a period of 2 years, based on a mean of 3.7 intakes per week for the eMBCT of the Helen Dowling Institute in 2011. An estimated attrition of 30% of the participants during both experimental interventions and 15% during the minimal intervention control condition [51] would leave us with 77 participants in each experimental group and 94 participants in the control group at T2. Again, we expect a dropout rate of 30% during the second semester. Such a dropout would leave a total of 110 participants completing each experimental intervention. Ten percent of the participants may have to be excluded from the analyses because of recurrence or diagnosis of metastasis. That would result in 198 participants that complete the full trial. We expect that this number will be enough for testing the 6 mediating factors or effect predictors: A classical, conservative power calculation (analysis of variance for testing 6 mediators or effect predictors with an intermediate effect size (f2=.08), corrected according to Bonferroni (alpha=.05/6), and at a power of .80 [81]) would result in approximately 254 participants being needed. We expect that the actual power when including 198 participants, and not the required 254 participants, will be great enough to detect up to 6 mediators or effect predictors with the use of Bayesian statistics [76]. Bayesian statistics allow analysis on small sample sizes [76,77], as more power can be generated with the use of prior information which is incorporated in the model that is being tested. Various papers describe comparisons between traditional null hypothesis testing and Bayesian estimation [82-85]. For this study, prior knowledge is available for many parameters, such as the effects of mindfulness in cancer survivors [23,25,66,86] and the role of working alliance in online interventions [87]. Examples of these methods can be found in both applied psychology and social science articles [88-92].

##### Missing Data Handling

Missing data will be analyzed considering their pattern and randomness following guidelines proposed by Schafer and Graham [93]. Bias due to systematic missing data will be managed according to guidelines proposed by Asendorpf et al [94].

##### Descriptives

Quantitative analyses will be conducted on an intention-to-treat basis. A flow diagram following the CONSORT guidelines will be included. Descriptive statistics will be calculated and presented. Independent samples’ t-tests and χ^2^ tests will be performed to check for baseline differences between the respective experimental conditions and the control condition with respect to demographic variables (eg, family status, age, gender, and level of education), time since end of treatment, and baseline levels of the outcome variables. If we find statistically significant differences in the mean of fatigue severity across baseline descriptives, dummy variables will be added to the model as covariates to control for these differences.

#### Core Analysis

##### Effectiveness

###### Overview

Five steps will be taken to evaluate the effectiveness of both interventions, which are explained here in a generic way. The specific hypotheses on the effectiveness of the interventions in our study are shown in Textbox 1.

**Hypotheses on effectiveness.**

*Primary outcome*

In both experimental conditions:

Fatigue severity

- decreases during the intervention, and
- remains decreased after 6 months.

After 6 months, fatigue severity has decreased significantly more compared to the control condition.

After 6 months, more participants show a clinically relevant reduction of fatigue severity compared to the control condition. A patient is considered clinically improved if he or she has a reliable change index of more than 1.96, according to the reliable change index, and the end score has to be within the normal range, that is a score < 1 standard deviation above the mean of a normative group [94] (ie, a score < 30.4 on the CIS fatigue severity subscale [51]).

*Secondary outcomes*

For both interventions we expect that:

- After 6 months, mental health and work ability have improved more than in the control condition.
- After 12 months, fatigue severity, work ability, and mental health remain improved in both intervention groups.
- Improvements in mental health and work ability after 6 and 12 months are related to reductions in fatigue severity.

Return to work and reduced absenteeism will be studied as explorative outcomes.

###### Step 1

Overall effectiveness will be tested in an intention-to-treat analysis by a multiple group latent growth model [79] using data from the first semester. This technique allows individuals to have an individual growth trajectory over time and compensates for missing data in an elegant way.

**Figure 4:**
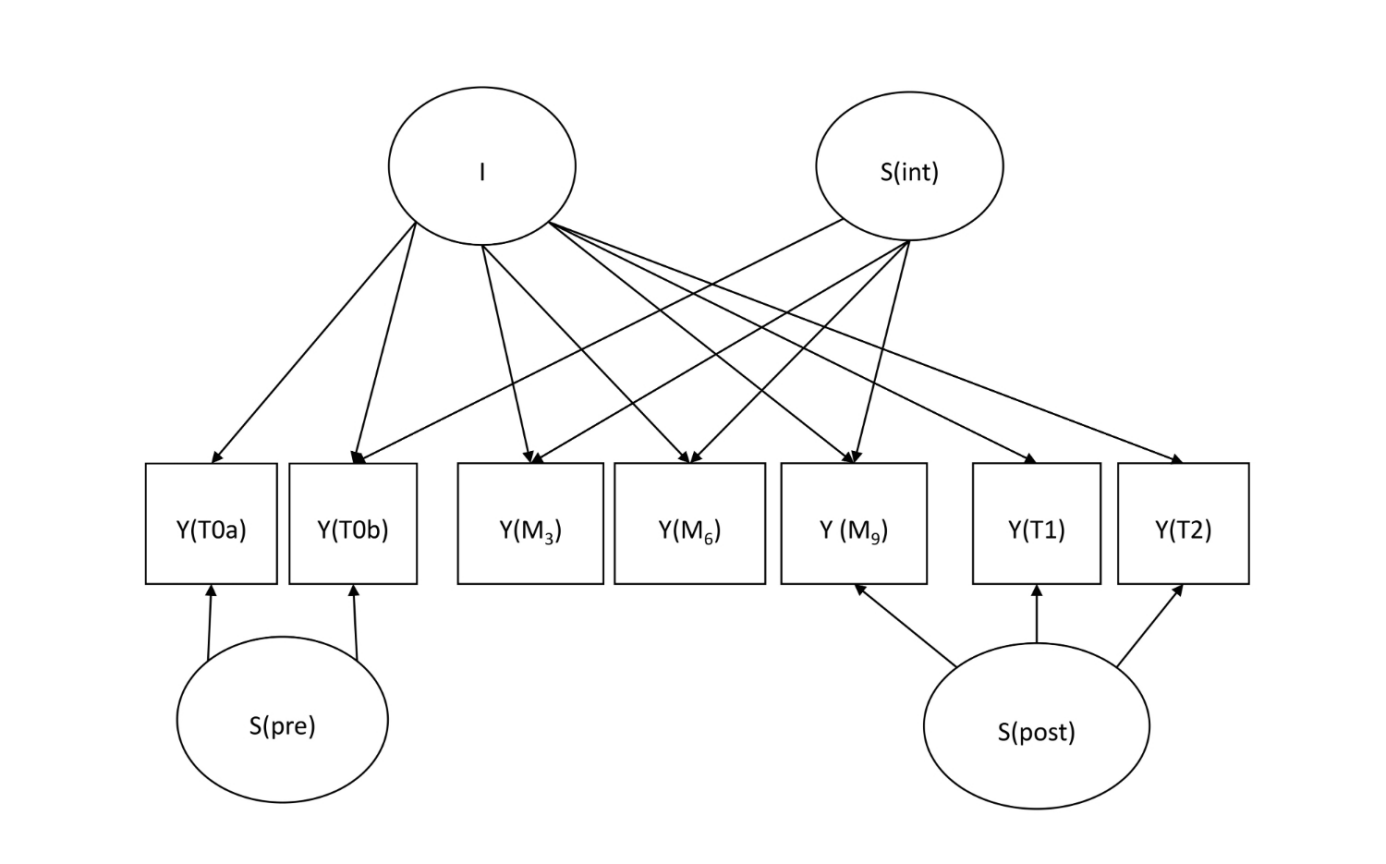
Simplified representation of a piecewise linear latent growth model, with latent intercept factor (I), latent slope factors preintervention (S(pre)), during the intervention (S(int)), and postintervention (S(post)), and 7 indicators Y. Error terms, correlation coefficients, and covariances are left out.

Since different growth patterns are expected for the pre-intervention period, the intervention period, and the post-intervention period, we will apply piecewise growth modeling so that a slope factor will be estimated for each of the 3 periods (Figure 4). Initial intercepts will be configured to represent the T0b score. This intercept and the pre-intervention slope factor will be constrained to be equal between all 3 conditions (and this assumption will be checked), whereas the subsequent slope factors will be estimated separately for the 3 conditions.

The fit of the piecewise model will be compared with a quadratic model. In the quadratic model, the entire first semester is modeled with 1 slope factor and 1 quadratic factor for each of the 3 conditions and an intercept that represents T0b and is constrained similarly to the piecewise model.

Both models will be run both with and without using time-varying loadings in order to check whether corrections should be made for differences in timings between the questionnaires. Growth factor estimates and model fits for all 4 models will be reported (Table 5).

Neither the participants nor the researchers (FBE and MW) are blinded to allocation. Therefore, an independent statistician (RvdS) who is blind to the allocation will test the primary hypothesis.

The same procedure will be followed for the secondary outcomes, except that the initial intercept of mental health will represent T0a, rather than T0b, because T0b does not include an assessment of mental health.

Results of frequentist analyses will be reported by *P*-values (significant in case <.05) and with 95% confidence intervals. Parameter estimates of models by means of Bayesian estimators will be reported with 95% central credibility intervals.

**Table 5:** Growth factor estimates of 4 different latent growth models.

	Fixed loadings	Time-varying loadings
Piecewise, linear	Mean: I, S(pre), S(int), S(post) Variance: I, S(pre), S(int), S(post)	Mean: I, S(pre), S(int), S(post) Variance: I, S(pre), S(int), S(post)
Quadratic	Mean: I, S, Q Variance: I, S, Q	Mean: I, S, Q Variance: I, S, Q

###### Step 2

The effect size of both experimental interventions will be calculated according to recommendations in Feingold (2009) [95] for both primary and secondary outcomes.

###### Step 3

The proportion of participants who make clinically relevant progress on the primary outcome will be calculated for all 3 conditions; again, in an intention-to-treat analysis. Percentages and standard deviations of the reliable change index will be presented.

###### Step 4

A latent growth model will be built of the primary outcome, in which the outcome measures that have been measured at T3 will also be included, as distal outcomes of changes in the primary outcome during the first semester.

###### Step 5

A growth mixture model (GMM) will be used to further explore differences between individuals, and more specifically to identify subpopulations (latent classes) with homogeneous growth trajectories of the primary outcome within the experimental groups. The Bayesian information criterion will be used for model selection [96].

If convergence considerations allow, this model will be adjusted to allow covariance of the growth factors in order to acknowledge individual variation around the estimated growth trajectories. The trace plots will be inspected to check whether the models have converged to global solutions and a set of diverse starting values will be used. For more information on these analyses, we refer to an introduction to GMM and latent class growth analysis by Jung and Wickrama [97] and examples of similar analyses in the field of Internet interventions [98] and cancer patients [41].

##### Mediators

###### Overview

The analysis of the mediators of the experimental conditions can be roughly subdivided into two steps: first analyze the primary factors individually for their longitudinal correlations with the outcome (Step 6), then combine the relevant factors in a multivariate analysis (Step 7). The specific hypothesis on the mediating factors of the interventions in this study are shown in Textbox 2.

###### Step 6

For analyzing the mediators of the experimental conditions, first we want to see whether there is a correlation between the growth trajectories of our outcome parameter and the potential mediator over time. The hypotheses considering mediators are shown in Textbox 2. The combined data from the participants in the first semester and data from the preferentially assigned participants in the second semester will be used.

**Figure 5:**
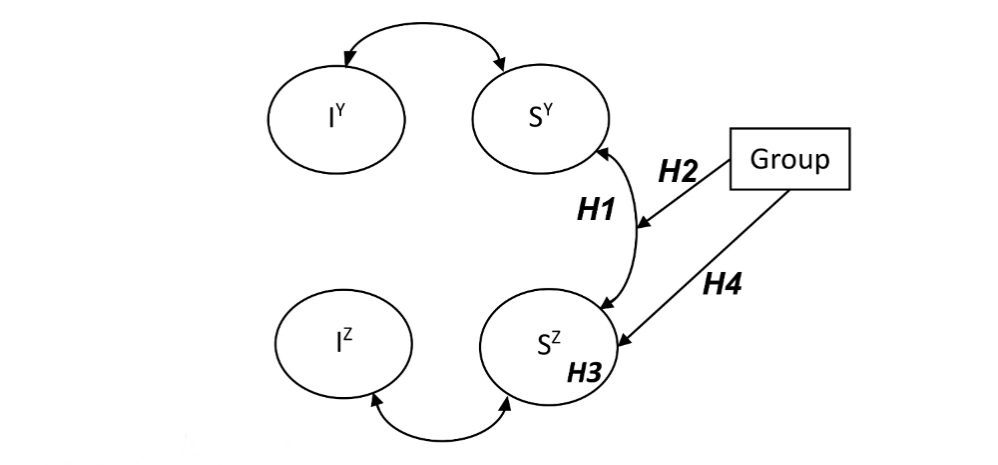
Simplified representation of a correlated growth model in which Iy and Sy represent the intercept and slope factors of the latent growth model of the outcome parameter, and Iz and Sz represent the latent growth factors of the mediator. H1-4 represent the 4 subhypotheses of Step 6. All indicators have been left out for clarity.

The following subhypotheses will be tested for each primary mediator (these are also shown in Figure 5):

1. Is the growth of the primary outcome (Sy) for the entire study population correlated with growth of the potential mediator (Sz)?
2. Is such correlation independent of group?
3. Does the potential mediator change over time in the specific group, so is the slope factor (Sz) substantially unequal to zero?
4. Is the slope factor in the specific group substantially greater than the slope factors in the other groups?

In these 4 subhypotheses, the first is congruent with testing the “conceptual theory” in classical mediation analysis, and subhypothesis 3 with testing the “action theory.” If subhypotheses 1-4 all are true, the factor will be considered a specific mediator for that intervention. If subhypotheses 1, 2, and 3—but not 4—are true, the factor will be considered a general mediator for fatigue severity. If either subhypothesis 1 (conceptual theory) or 3 (action theory) is false, the factor will not be considered a mediator.

**Hypotheses on mediators.**

We expect that, for AAF, increasing mean cumulative daily physical activity and reductions of daily physical activity decline are specific mediators.

We expect that, for eMBCT, developing mindfulness skills is a specific mediator.

We expect that sleep quality, working alliance, sense of control over fatigue, credibility, and expectancy are generic mediators for both e-therapies.

The mediating role of the following factors will be explored: number of sessions completed, changes in causal attributions [99], decreased catastrophizing thoughts about fatigue [8], decreased fear of cancer recurrence [100,101], changes in perceived activity [102], increased self-efficacy on physical activity [103].

###### Step 7

The next step in studying potential working mechanisms is a single-step, multiple-mediation analysis using structural equation modeling [104-106]. By estimating such a model, we expect to obtain a comprehensive model for all the working mechanisms of the intervention. It should be noted that this model assumes that an intervention works in the same way for all participants in a particular group [107]. Again, data from both semesters will be used.

**Figure 6:**
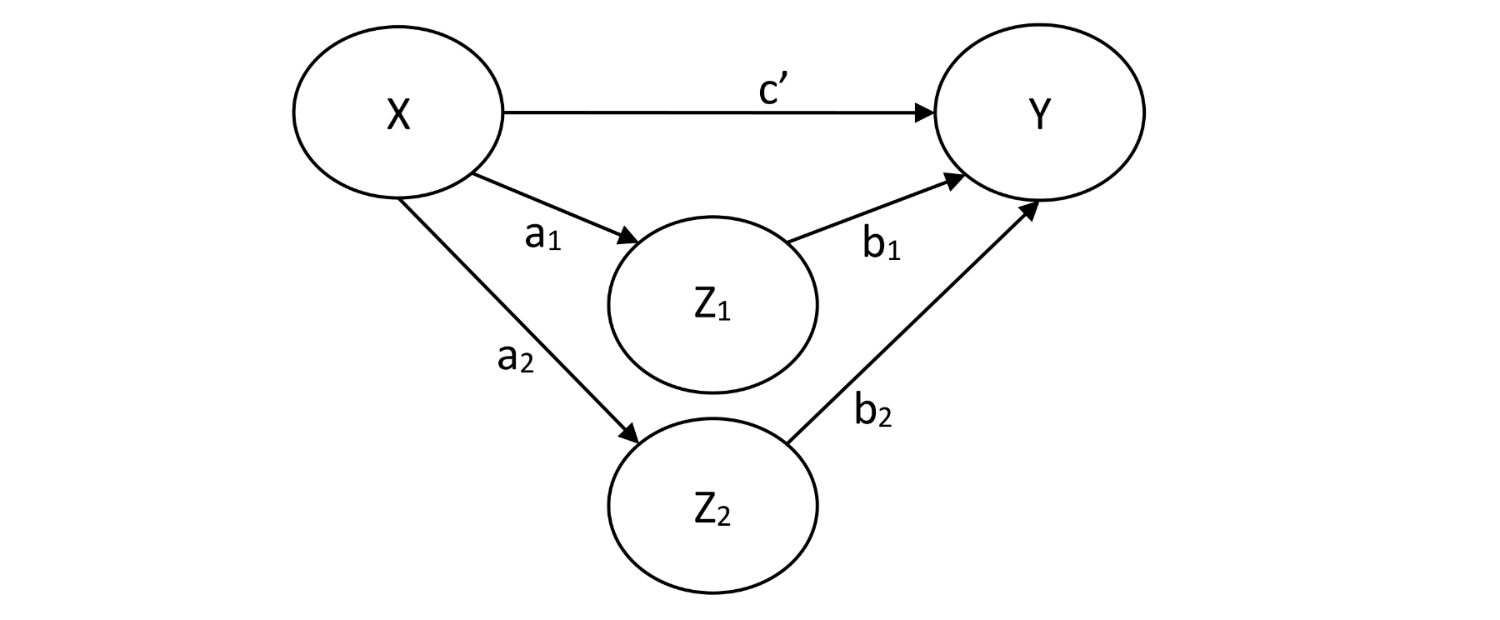
Multiple mediation model with independent variable (X), dependent variable (Y), and 2 mediators (Z). Direct effect (c’) and indirect effects (through a x b) are shown.

A separate model will be tested for each intervention. Each model will have the following paths (Figure 6), where X=independent variable (1/0 for specific intervention vs control group), Y=outcome variable (difference score T2-T0b of the primary outcome measure), and Z=mediator:

- a: X regressed on Z.
- b: Z regressed on Y;
- c’: direct effect of X on Y.

For each experimental intervention, the starting model will consist of all the significant primary mediators of Step 6 that have also been assessed in the control group. In other words, the factors that have shown to be mediators in the correlated growth model will be the starting point for this model. The models will then be complemented with the secondary mediating factors described in Textbox 2. Mediating factors for which the indirect effect (a × b) is insignificant will be removed stepwise, after which a final model will be created.

Model fit, standardized path coefficients—including indirect effects—and the total effect of at least the first and final models will be reported with 95% confidence intervals.

##### Effect Predictors

###### Overview

Two complementing approaches for analyzing the effect predictors are addressed in steps 8 and 9 of this analysis plan. The specific hypotheses on the effect predictors for both interventions in this particular study are shown in Textbox 3.

**Hypotheses on effect predictors.**

We expect that for AAF, low perceived physical activity predicts reduction of fatigue severity.

We expect that for eMBCT, low perceived concentration, previous experience with mediation exercises, high perceived life threat from cancer, and a high education level predict reduction of fatigue severity.

In general, we expect that low perceived social support, longer time since last treatment, suffering from more comorbidities [108], and having strong somatic attributions [57] predict small effects on fatigue severity in both interventions. We expect that high sense of control and good sleep quality predict large effects in both experimental conditions.

Other factors will be included for explorative research.

###### Step 8

To find out which participants benefit most from each intervention, the final model of fatigue severity of Step 1 will be extended with potential effect predictors (Textbox 3) that load on the latent growth factors “linear slope” (the “post randomization” linear slope in case of the piecewise model) and, if applicable, “quadratic slope.” As the regression coefficients of all potential effect predictors on the development of fatigue severity will be freely estimated across the 3 intervention groups, this is also called a moderation effect of intervention.

Factor loadings of all hypothesized effect predictors will be reported. Those with the highest loadings will be compared between the conditions in order to find differential effect predictors.

###### Step 9

To identify common effect predictors of homogeneous subpopulations within the heterogeneous population, rather than identifying effect predictors for individual growth patterns, the final step will consist of regressing predictors on latent classes. Therefore, the final model of Step 5, the GMM, will be extended. Again, several potential effect predictors will be regressed onto this model, but this time on the latent class factor, instead of on the latent growth factors. The 3-step procedure proposed by Vermunt [109] will be used for model selection. This step will be carried out separately for each experimental condition.

Factor loadings of all hypothesized effect predictors will be reported. Those with the highest loadings will be compared between the conditions in order to find differential effect predictors.

## Results

Recruitment for the trial started in March 2013 and is expected to continue until April 2015. No major changes have been made to the protocol. However, due to an error in the randomization algorithm between January 14, 2014, and July 15, 2014, allocation was dependent on the number of participants who were allocated at once. This in turn was completely random. Consequently, 10 participants were allocated directly to the AAF group; 4 other participants were divided equally between the 2 experimental interventions; and 15 accounts (of which, 1 was a dummy account) were equally divided between the 3 groups. None of the researchers were aware of this error, as this allocation could very well have simply been the result of the “roll the dice” scenario that should have been applied. How many participants were allocated at once was not the subject of the researchers’ decision-making. Therefore, we argue that allocation has still been random and, accordingly, data for all considered participants will be processed as originally planned.

At the time of this writing in January 2015, 269 patients have registered at the project website. Of these, 111 have been officially included in the study, 50 were excluded from participation, and 35 withdrew before their eligibility was checked. The remaining patients are still in the enrollment phase. The main reason for exclusion so far has been a score lower than 35 on the CIS fatigue severity subscale (60%). Furthermore 11% did not meet the psychiatric stability requirement, 8% were younger than 18 at the time of cancer diagnosis, and 8% were still receiving cancer treatment.

Current group sizes as of January 2015 for participants in the first semester are 36 (AAF), 24 (eMBCT), and 32 (control). However, 19 participants have not yet been randomized. Initial responses to the primary research question are expected to be available by the end of 2015.

## Discussion

### Principal Findings

This paper has described the design, hypotheses and analysis plan of a randomized controlled trial in order to study the effectiveness, mediation, and effect predictors of 2 Internet-based interventions for CCRF. Although recruitment and inclusion have already started, publishing the analysis plan is of great value because it will help to prevent outcome reporting bias [110] and adds validity information to the final studies [111].

By using multiple assessments during the intervention, the proposed trial design is suitable for studying the chronological development of both potential mediators and fatigue. That has 2 main advantages. Firstly, the data will be suitable for analyses that allow for variation in the individual fatigue trajectories. We do not expect that either of the interventions that have been included in the trial will be beneficial for all participants: our study sample will be highly heterogeneous considering for example tumor and treatment types. Therefore, the analyses on individual growth trajectories can acknowledge that expectation and test that hypothesis. This will substantiate the interpretation of the results on effectiveness and will be an important first step in identifying what works for whom. Secondly, this study design enables us to use a fully longitudinal mediation analysis, at least for the most important factors, rather than using indirect effects analysis in cross-sectional mediation analysis.

Another important feature of the proposed design is that by comparing 2 different interventions with an active control group, therapy-specific elements of the interventions can be distillated from the data acquired during this trial. This advantage counts for both the effect predictors and the mediators. Knowledge about such differentiating factors can and should be used to better inform patients with CCRF and to improve allocation of patients with CCRF to suitable interventions. As a result, an increase in the overall effectiveness of relevant interventions can be established.

In this paper, we have presented the trial design, our hypotheses, and a detailed analysis plan. In accordance with good clinical practice, and to avoid outcome reporting bias, this paper was submitted before any of the data was analyzed. All methods are now openly predetermined, therefore any future publication describing this trial can be valued reliably on its quality.

### Limitations

A limitation of the current paper is that for most instruments, this paper does not include information on its properties or a thorough rationale for its choice. More extensive information on the actual instruments will be reported in subsequent papers on the results of the various research questions posed in this trial.

### Conclusions

Given the growing number of patients suffering from CCRF, the availability of effective Internet interventions potentially strengthens current health care for this population substantially. We have proposed a design to study 2 Internet interventions in order to gain insight into their effectiveness, mediators, and effect predictors, which fully acknowledges differences between individual patients and differences in the way they respond to each intervention. Results on the effectiveness and mediators will give useful information for improving both the quality and availability of such interventions. Also, identifying effect predictors for positive intervention effects will improve the referral of patients to relevant interventions. By presenting our hypotheses and analytic strategy before completion of data collection, this paper is a first step in carefully reporting on this comprehensive trial.

## Appendix

Multimedia Appendix 1 Picture of the ProMove accelerometer and mobile phone app with feedback.

Multimedia Appendix 2 Additional screenshot of the therapist Web portal for ambulant activity feedback therapy (Dutch).

Multimedia Appendix 3 Additional screenshot of the patient Web portal for eMBCT (Dutch).

Multimedia Appendix 4 Example of an information letter for the minimal intervention control condition (Dutch).

Multimedia Appendix 5 CONSORT E-HEALTH checklist V1.6.1 [112].
