# Supplementary figures and images for: Effectiveness, Mediators, and Effect Predictors of Internet Interventions for Chronic Cancer-Related Fatigue: The Design and an Analysis Plan of a 3-Armed Randomized Controlled Trial
Source: JMIR Res Protoc. 2015 Jun 23;4(2):e77. doi: 10.2196/resprot.4363 (PMC4526958; doi:10.2196/resprot.4363)

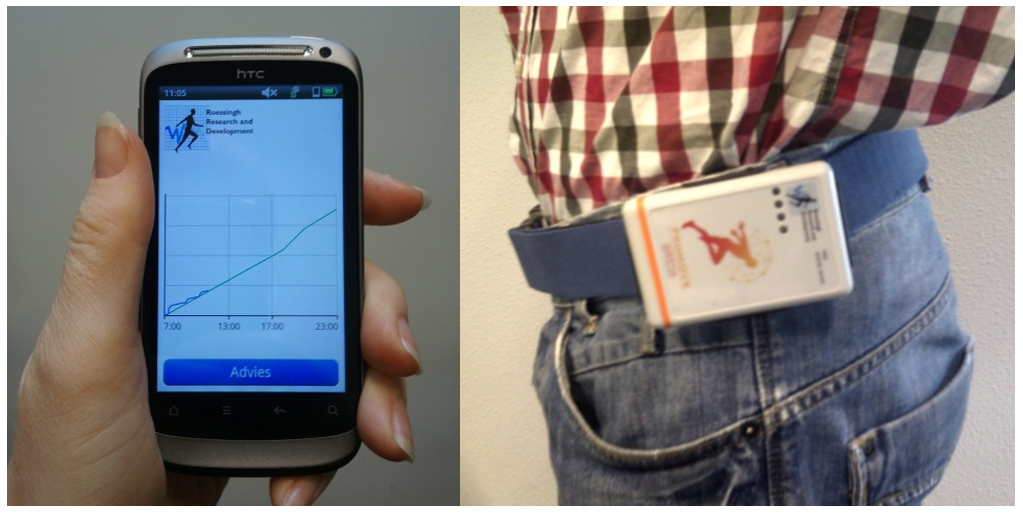

Supplement: Multimedia Appendix 1 [file resprot_v4i2e77_app1.PNG]

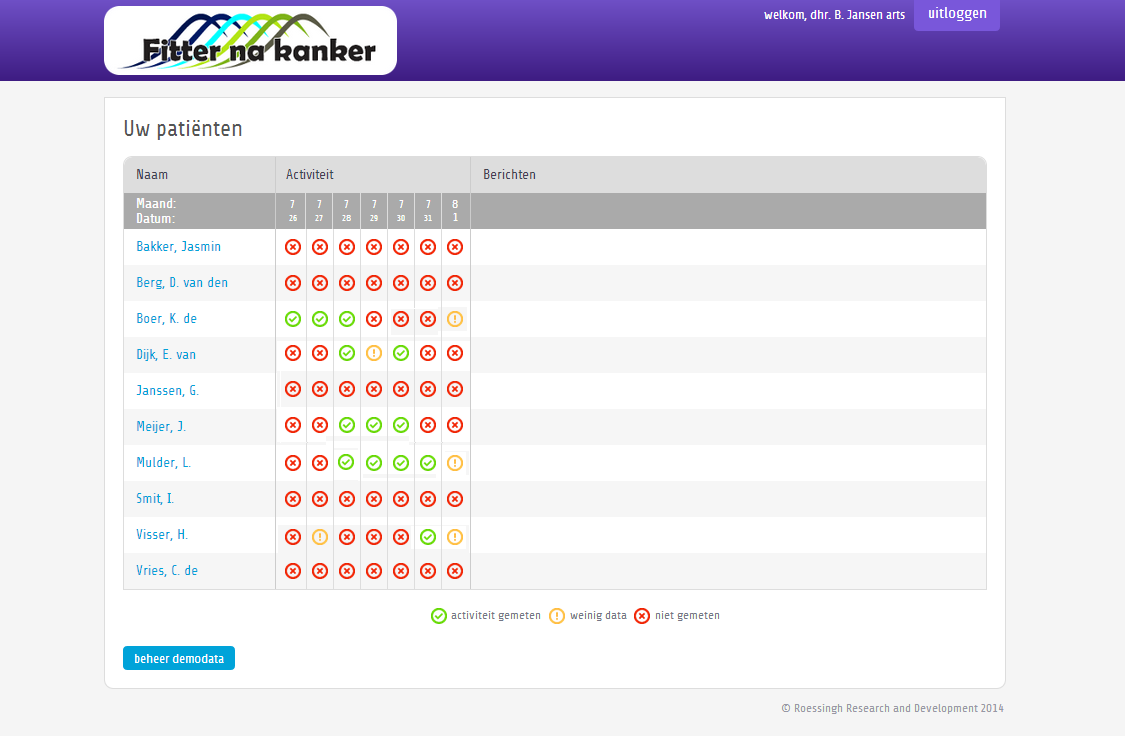

Supplement: Multimedia Appendix 2 [file resprot_v4i2e77_app2.png]

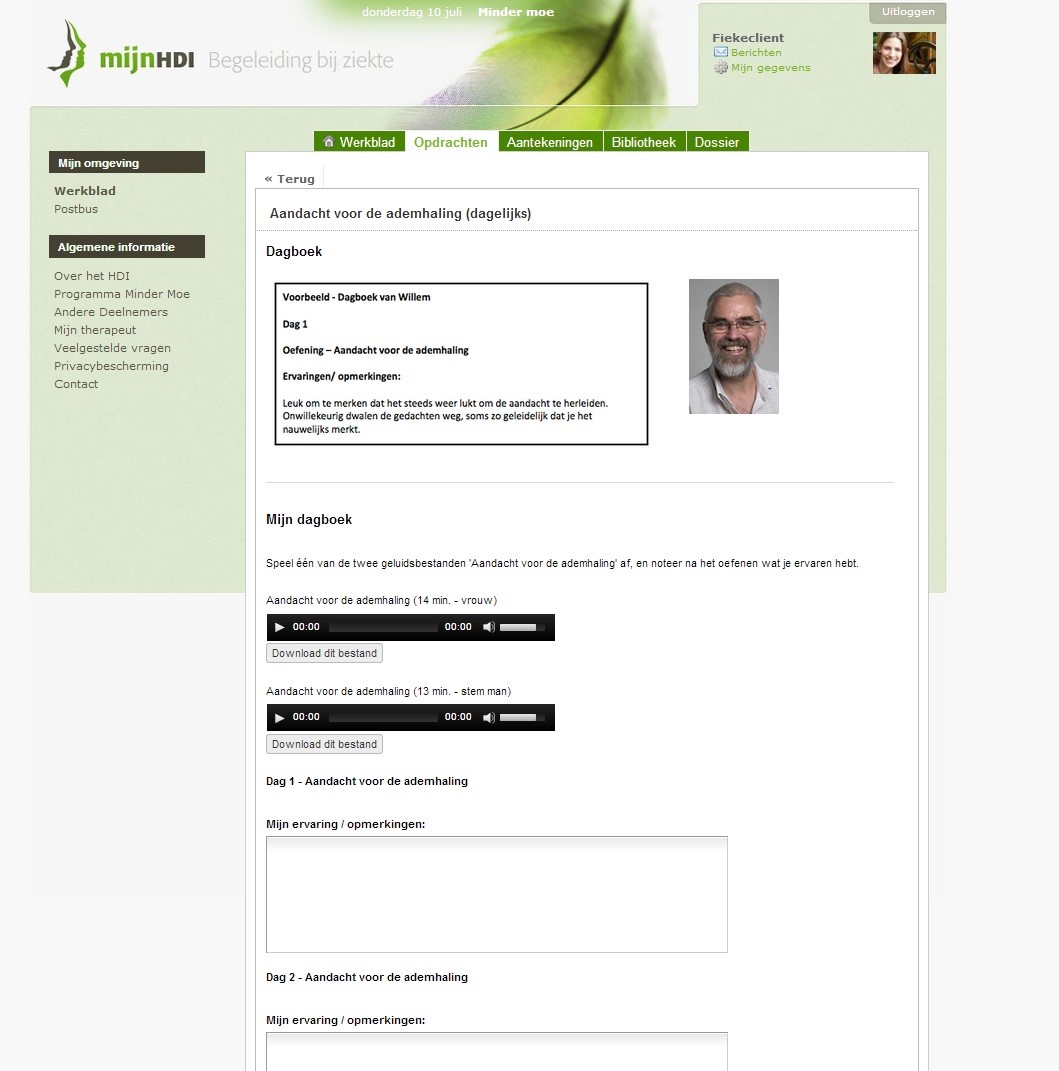

Supplement: Multimedia Appendix 3 [file resprot_v4i2e77_app3.jpg]

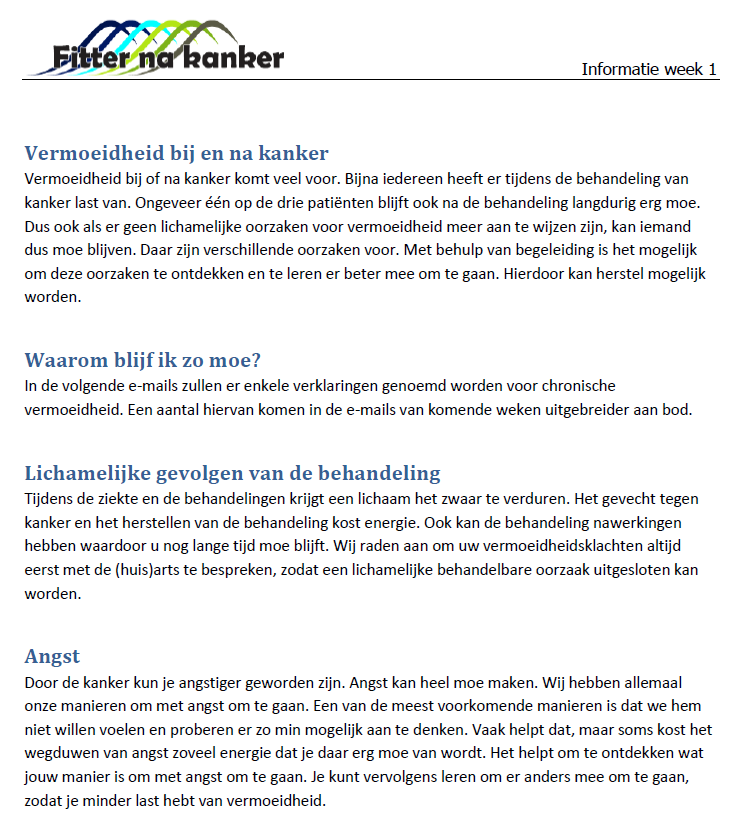

Supplement: Multimedia Appendix 4 [file resprot_v4i2e77_app4.png]
